# Phenolic Profiles, Antihyperglycemic, Anti-Diabetic, and Antioxidant Properties of Egyptian *Sonchus oleraceus* Leaves Extract: An In Vivo Study

**DOI:** 10.3390/molecules28176389

**Published:** 2023-09-01

**Authors:** Nesrein S. Salim, Mohamed Abdel-Alim, Huda E. M. Said, Mohamed F. Foda

**Affiliations:** 1 Department of Biochemistry, Faculty of Agriculture, Benha University, Moshtohor 13736, Egypt; nesreen.salem@fagr.bu.edu.eg (N.S.S.); mohamed.abdelalem@fagr.bu.edu.eg (M.A.-A.); 2 Clinical Pathology Department, Faculty of Medicine, Zagazig University, Zagazig 44519, Egypt; hesaid@medicine.zu.edu.eg; 3 College of Life Science and Technology, Huazhong Agricultural University, Wuhan 430070, China

**Keywords:** *Sonchus oleraceus*, DPPH, hyperglycemic, total phenolic, and flavonoid

## Abstract

This study aimed to investigate the phenolic and antioxidant properties of Egyptian *Sonchus oleraceus* leaves extract (SOE) while comparing the antihyperglycemic efficacy of SOE with that of conventional medicines (glibenclamide) in vivo as a substitution for insulin-deficient patients. Total phenolic (TPC) and flavonoid contents (TFC) in SOE contributed around 127.66 ± 0.56 mg GAE/gm as gallic acid equivalent (GAE) and 74.80 ± 0.55 mg QE/gm as quercetin equivalent (QE). SOE also showed significant DPPH scavenging activity at 43.46%. The presence of five phenolic and six flavonoid compounds in SOE was discovered by HPLC analysis. For the in vivo assay, 42 rats were distributed into six groups (7 Wister albino rats each). The standard control group was fed a basal diet. While the 35 rats were induced with a single dose of 100 mg kg^−1^ body weight (b.w.) alloxan, then treated orally with glibenclamide (GLI) at 10 mg kg^−1^, 100, 200, and 300 mg kg^−1^ SOE (positive control group) for 56 days of routine gastric oral gavages and compared to the effects of GLI, the treatment of SOE 200 and 300 mg kg^−1^ in diabetic rats for two months dramatically decreased blood glucose, total lipid, total cholesterol, and low-density lipoprotein cholesterol (LDLC) while boosting high-density lipoprotein cholesterol (HDLC) levels and improving liver and kidney functions. The histological assay revealed that the SOE 300 mg kg^−1^ treatment significantly improved the pancreas tissues, implying the potential application of Egyptian SOE as a diabetes treatment.

## 1. Introduction

Inadequate insulin causes the metabolic syndrome known as diabetes mellitus, which leads to hyperglycemia and insufficient cell glucose uptake. Deity, aging, sedentary lifestyles, and growing urbanization are all thought to play a role in the increased diabetes mellitus [1]. Cerebrovascular disease, peripheral vascular disease, and coronary heart disease are among the conditions that can be influenced by the effects of diabetes on both microvascular and macrovascular systems. These effects can give rise to complications such as diabetic neuropathy, diabetic retinopathy, and diabetic nephropathy. Additionally, gastroparesis, diarrhea, infertility, infections, skin infections, cataract, glaucoma, and periodontal disease can be caused by macrovascular problems [2]. 

Eating veggies has been linked to decreased infectious illnesses spurred by oxidative stress because of their phenolic antioxidants [3]. *Sonchus oleraceus* L. (Family: Asteraceae), also known as sowthistle, is a leafy vegetable that can be eaten directly. It is often consumed in Mediterranean countries [4]. Traditional healers have used the aerial parts of this plant in Cameroon to treat diabetes [5], stomach pain, hepatitis, infections, inflammation, headaches, general nuisance, and rheumatism [6]. Numerous pharmacological properties of *S. oleraceus* (SO), including antioxidant, anti-diabetic, and anti-inflammatory actions, have been previously reported [5,7,8].

Phenolic composites, for instance, phenolic acids and flavonoids, have been related to various biological activities in edible and non-edible plants, including antioxidant activity. Crude extracts of herbs, vegetables, and other plant materials superior in phenols are becoming popular in the food industry because they hinder the oxidative demolition of lipids, enhancing food quality and nutritional value. As the future trend turns toward functional food with specific health effects, the antioxidant elements of plant materials are becoming increasingly significant in the preservation of health and protection against coronary heart disease and cancer. Phenolic acids may provide health advantages by lowering the risk of metabolic syndrome and the difficulties associated with type-2 diabetes. However, different phenolic acids have additional biological properties, and restriction remains to be understood about the mechanisms by which they might play a role in disease prevention. Polyphenols are not essential nutrients, but nutrition research has shown that long-term and acute intakes can improve weight management and chronic illnesses such as CVD, obesity, type-2 diabetes, certain malignancies, and cognitive function [9,10,11,12,13].

More interestingly, polyphenols’ effects are inextricably linked to their bioavailability. Only about 5% to 10% of total dietary polyphenol intake is thought to be absorbed directly through the stomach and/or small intestine, with the remainder passing through the colon and being transformed by the microbiota [14,15]. Polyphenols experience phase I and phase II metabolism in the liver after absorption (sulfation, glucuronidation, methylation, and glycine conjugation). Polyphenol metabolites produced by hepatic metabolism, among other sites of bioactivity, may interact with adipose tissue, the pancreas, muscle, and the liver. Many phenolic compounds in plant foods have recently attracted much attention due to their ability to influence starch digestibility and potent antioxidant properties [16]. These compounds have also been reported to promote health benefits by reducing the risk of diabetes and its complications such as catechins, procyanidins, caffeic acid, chlorogenic acid, p-coumaric acid, berberine, quercetin, o-coumaric acid, ferulic acid, protocatechuic acid, quercetin, and gallic acid [17,18,19,20,21]. Quercetin is a vital polyphenolic flavonoid present in vegetables and fruits, and its role in promoting health has been demonstrated previously [22,23]. 

Furthermore, its role as an anti-inflammatory, antioxidant, and anti-angiogenesis was proven in the previous study [24]. It was reported that quercetin had a role in restoring antioxidant enzyme activity in kidney tissue of Diclofenac-treated rats. Furthermore, in the presence of quercetin, Diclofenac could not enhance the expression of pro-inflammatory cytokines, advocating that quercetin may have anti-inflammatory potential [25]. Its role in cancer has been documented by modulating various biological activities [26,27,28,29,30].

In this study, conventional assays were employed to assess the antioxidant properties of S. oleraceus using various extraction solvents. These assays included evaluating the free radical scavenging activity through DPPH assay and determining the total phenolic and flavonoid content. We also aimed to assess the potential treatment effect of the *Sonchus oleraceus* extract (SOE) against hyperglycemia in albino rats to determine whether SOE can be utilized as a natural antioxidant source for diabetes mellitus in vivo. 

## 2. Results

### 2.1. The Extraction of Sonchus oleraceus Extracts (SOEs)

The extraction yields of different SOEs are reported in Table 1. Based on the results, total ethanolic extract (E.E.) produced the highest extraction yield of 33.4%, while the lowest yield was chloroformic (C.F.), 5.4%. According to Dhanani et al. (2017), the yield amount indicated the extraction conditions’ effects. Thus, the variations in the extraction yields were due to the differences in the solvents’ polarity and the extraction method, which directly impacted the biological activities of the final extracts [31,32,33]. As the most polar solvent, water was the most efficient solvent in extracting the phytochemical constituents from the plant. The combination of water and organic solvent (ethanol), which also had high polarity, further enhanced the extraction efficiency. Supported by the previous study that showed a high polar solvent resulted in a more significant percentage of the yield than a less polar solvent [34]. 

The total phenolic and flavonoid results are presented in Table 1, respectively. E.E. contained the highest phenolic content, 127.66 ± 0.56 mg GAE/gm, followed by A.E., 105.5 ± 0.24 mg GAE/gm. The lowest phenolic content was obtained from P.T. 61.25 ± 0.76 mg GAC/gm. Similarly, E.E. and A.E. exhibited the maximum amount of total flavonoid with 74.80 ± 0.55 mg QE/gm and 37.13 ± 0.75 mg QE/gm, respectively. Additionally, the ethanolic extract demonstrated the maximum antiradical activity in DPPH at 43.46%, followed by the A.E. at 37.82%. Therefore, the ethanolic extract was used in further biological experiments. The data coordinated with Jie Yin et al. reported in 2007 [35].

### 2.2. Identification of Some Antioxidant Components of SOE Extracts by HPLC

Depending on their bioavailability, which is highly determined by their degree of polymerization, these natural polyphenol fractions are significant in human health. The phenolic compounds profiling was evaluated to assess the SO extract’s biological activities. Retention time (RT) was used to determine each peak. Eleven compounds of polyphenols (phenolic and flavonoid fractions) were identified in Figure S1 using HPLC, and their concentrations were evaluated, as shown in Table 2. The main identified phenolic compounds in SOE extract were Catechin and Chlorogenic acid, with a concentration of 37.93 and 28.2, respectively. Likewise, data show that SOE extract contains six flavonoid compounds. Hesperidin, Vanillin, Naringin, and Quercetin were the major compounds, with a concentration of 24.79, 16.58, 11.30, and 4.29. The declared data are consistent with the data given in [34,36]. 

Furthermore, the ethanolic extract was chosen for the biological assay because it is known to contain more potent bioactive phytoconstituents than other solvent extracts and because further pharmacological studies with SOE extract will be conducted [37]. 

### 2.3. Biological Evaluation

Serum glucose, lipid profile, liver, and kidney function were assessed 56 days into the investigation. There were no discernible variations in beginning body weight between treatments for any groups at zero time. The final body weight of diabetic rats significantly increased linked to the healthy control group, according to the findings in Table 3. After 56 days of treatment, rats with diabetes and normal rats had improved final body weights thanks to SOE (100, 200, and 300 mg kg^−1^). The given extract’s phenolic and flavonoid components may be responsible for this. Compared to the control groups, the body weight of the diabetic rats treated with glibenclamide (10 mg kg^−1^) was significantly lower. These findings support those of [38,39] and tend to be consistent with previous World Health Organization studies, which stated that diabetes mellitus is frequently characterized by rapid and severe weight loss and subsequent exhaustion that is difficult to reverse [40].

#### 2.3.1. Effect of Treatment with SOE Extract and Glibenclamide (GLI) on Blood Glucose in Negative Control and Positive Control (Diabetic) Groups

Alloxan specifically kills the insulin-secreting cells in the pancreas, leaving less active cells and causing diabetes. A significant reduction in body weight characterizes alloxan-induced diabetes. The loss or degradation of structural proteins known to contribute to body weight is the cause of this drop. The elevated glucose levels in animals show this impact [41]. Fasting blood glucose results are presented in Table 4. A significant (*p* < 0.05) augmentation in fasting blood glucose levels in the diabetic group (405 mg dL^−1^), as compared with the negative control (104.3 mg dL^−1^), was recorded after 56 days. More significantly, the administration of SOE (200 and 300 mg kg^−1^) and/or glibenclamide (10 mg kg^−1^) in diabetic rats for 56 days reduced blood glucose levels by (138.7, 103.7, and 184.3 mg dL^−1^, respectively). The mentioned data are in harmony with those reported by [5].

#### 2.3.2. Effect of SOE Extract at Different Doses on Serum Lipid Profile

The serum lipid profile in normal diabetic rats is presented in Table 5. Diabetic rats had significantly (*p* < 0.05) higher serum levels of TC, TG, and LDL (group 2). Instead, HDL decreases statistically significantly compared to the negative control group (*p* < 0.05). These findings support [21], who found that streptozotocin-induced diabetic rats had significantly higher TC and TG levels and lower HDL levels. Since their levels fell compared to diabetic rats after receiving 200 mg kg^−1^ SOE and 10 mg kg^−1^ glibenclamide for 56 days, serum lipid profile (TC, TG, and LDL) improved (group 2). Compared to diabetic rats, group 5, treated with 200 mg kg^−1^ of SOE, showed a substantial (*p* < 0.05) rise in HDL levels (group 2). These findings support those of [5].

#### 2.3.3. Elevation in Hepatic and Renal Enzymes in SOE Extract and Glibenclamide-Treated Rats

Data presented in Table 6 show a significant (*p* < 0.05) increase in hepatic enzyme (AST, ALT, and TB) levels in diabetic rats control (group 2) compared with the negative control group. The increment in the activities of the hepatic enzymes may be attributed to the leakage from liver cells into the bloodstream, which in turn causes the hepatotoxic effects of alloxan, as stated by [42,43]. The obtained results of the impact of SOE on hepatic enzymes in diabetic rats (group 4,5,6) exhibited a significant ameliorate in hepatic enzyme levels Table 6 compared to control diabetic rats (group 2). 

A significant increment was noted in the serum urea, creatinine, and uric acid levels in diabetic rats control (group 2) compared with the negative control Table 6. Diabetes can damage the kidneys’ blood vessels, leading to abnormal renal function, represented by reduced glomerular filtration and a rise in serum urea and creatinine [44]. Data presented in Table 6 show a significant decrease in serum urea, creatinine, and uric acid levels in rats treated with SOE (300 mg kg^−1^) in diabetic rats (group 6) compared with diabetic control rats. These results favor treating hepatic and renal functions in diabetic rats treated with SOE extract (300 mg kg^−1^).

#### 2.3.4. Effect of SOE and Glibenclamide on Antioxidant Parameters in Diabetic Rats Compared to GLI, Negative Control, and Positive Control (Diabetic) Groups

The effects of the different extract concentrations on the Malondialdehyde (MDA) concentrations in plasma are summarized in Table 7. In general, the MDA concentration was significantly higher in the plasma of the diabetic rats (group 2) compared with a standard group and those receiving Glibenclamide. The concentrations in treated rats were significantly lower than the values for the untreated (*p* < 0.05), with SOE at 300 mg kg^−1^ as the most active. The catalase activity, SOD, GSH, and GST significantly increased in rats receiving SOE plant extracts 100, 200, and 300 mg kg^−1^ as compared with diabetic animals (group 2) (*p* < 0.05). The mentioned data are in harmony with those reported by [5], who studied the effect of hydroethanolic extract of SOE plant (150 and 300 mg kg^−1^).

#### 2.3.5. Histological Assay

**Figure 1:**
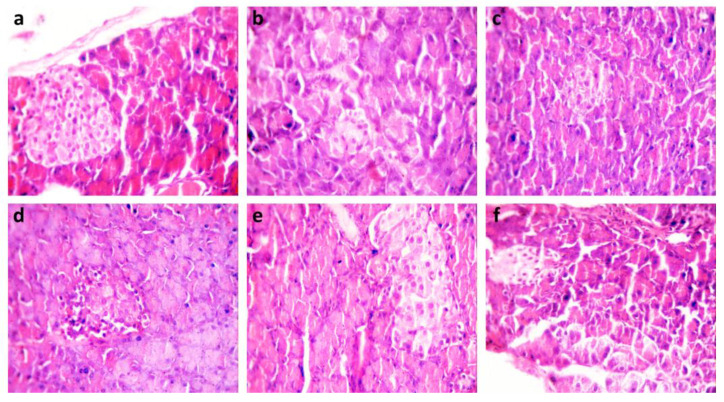
(**a**) Pancreas section from a rat without any treatment (G1) showing a normal structure of islets of Langerhans (H&E × 200); (**b**) Pancreas section from a diabetic rat (G2) displaying degenerative changes in pancreatic acini and islets of Langerhans (H&E × 200); (**c**) Pancreas section from diabetic rats treated with Glibenclamide at 10 mg kg^−1^ (G3) exhibiting a nearly normal histological structure with focal regeneration of islets of Langerhans (H&E × 400); (**d**) Pancreas section from diabetic rats treated with SOE at 100 mg kg^−1^ (G4) showing mild degenerative changes with few focal mononuclear cellular infiltrations (H&E × 100); (**e**) Pancreas section from diabetic rats treated with SOE at 200 mg kg^−1^ (G5) revealing focal segmentations and hyalinosis (H&E × 100); (**f**) Pancreas section from diabetic rats treated with SOE at 300 mg kg^−1^ (G6) displaying a normal histological structure of islets of Langerhans (H&E × 200).

In the negative control group, serial sections from the pancreas exhibited normal histological structure, with intact islets of Langerhans and a healthy acinar epithelium with well-preserved secretory granules (Figure 1a G1). However, in the pancreas tissues of alloxan-induced diabetic rats (G2), microscopic examination revealed degenerative changes in pancreatic acini and islets of Langerhans. Complete hyalinization with leukocytic infiltration or complete atrophy was observed (Figure 1b). On the other hand, rats treated with Glibenclamide 10 mg kg^−1^ (G3) showed nearly normal histological structure, with focal regeneration of islets of Langerhans (Figure 1c). In the groups treated with 100, 200, and 300 mg kg^−1^ of SOE (G4-G5-G6), the pancreas gradually improved the histological structure of the islets of Langerhans as presented in (Figure 1d–f), respectively.

## 3. Discussion

Natural compounds, as whole or specific active compounds of medicinal plants, play an essential role in the inhibition of pathogenesis, including diabetes, as quercetin has a role in the management of diabetes mellitus [45,46]. We evaluated the antioxidant, antihyperglycemic properties, and anti-diabetic potential of *S. oleraceus* using a variety of methods, including determining the total polyphenol content (TPC) and total flavonoid content (TFC) of the extract, assessing its capacity to scavenge free radicals and analyzing its impact on rat hyperglycemia. Higher quantities of polyphenols were found in the ethanolic *S. oleraceus* leaves extract, which also exhibited a better free radical scavenging action, a considerable ability to lower blood sugar levels, and a drop in oxidative stress indicators. By reducing oxidative stress brought on by disease, phenolic compounds—known to be produced by plants as a defensive mechanism against abiotic and biotic stresses—benefit people as well [47].

We used a significant amount of sugar to cause hyperglycemia in rats to test the extract’s potential to increase glucose production and determine its effects on hyperglycemia. We employed effectively 100, 200, and 300 mg kg^−1^ extract dosages. As shown in Table 4, all *S. oleraceus* dosages improved blood glucose levels compared to the diabetic group. These results align with earlier anti-diabetic investigations, which revealed appreciable drops in serum blood glucose levels in diabetic rats after therapy [5]—highlighting the potential therapeutic effects of Egyptian *Sonchus oleraceus* ethanolic extracts (SOE) as a treatment for diabetes. Our research findings indicate that SOE may be beneficial for diabetes management without causing adverse effects on the liver, kidney functioning, or lipid profile. This study involved comparing a negative control group, which displayed a normal histological structure of the pancreas, with alloxan-induced diabetic rats exhibiting degenerative changes. Interestingly, the treatment with Glibenclamide and SOE at a dosage of 300 mg kg^−1^ resulted in significant improvement in the pancreas’s histological features, suggesting its potential as a therapeutic approach for promoting pancreatic health.

Uncontrolled type-2 diabetes mellitus is known to raise triglycerides and LDL-cholesterol and reduce HD-cholesterol, all of which help to cause coronary artery disease [38]. In contrast to the healthy control rats in our study, alloxan-treated rats showed a significantly higher total cholesterol and triglyceride level as well as a lower HDL level. However, as shown in Table 5, diabetic rats given 200 mg kg^−1^ of *S. oleraceus* extract had a considerable decline in total cholesterol and triglyceride levels and increased HDL levels. Table 6 further shows that, compared to control rats, rats treated with alloxan had significantly higher levels of ALT, AST, urea, creatinine, and uric acid. Nevertheless, liver and kidney enzyme levels were significantly reduced in diabetic rats given 300 mg kg^−1^ of *S. oleraceus* extract.

The study also examined how *S. oleraceus* extract controls defense enzymes, which is essential for shielding biological components from oxidative damage. It is well-recognized that oxidative stress plays a role in the emergence of cardiovascular lesions [48] and that diabetes exacerbates these effects [49]. Superoxide dismutase (SOD) expression was found to be lower in type-2 diabetic individuals in several investigations. Following this, SOD activities were enhanced by treatment with *S. oleraceus* extract. The research also showed that the total amount of glutathione (GSH) in the livers of diabetic rats had significantly decreased. GSH has the power to lower the rate at which H_2_O_2_ is scavenged and to prevent the development of hydroxyl radicals, both of which lower the generation of internal radicals. In diabetic rats, administration of *S. oleraceus* extract raised hepatic GSH levels. Therefore, the reduced amounts of SOD and GSH probably helped to reduce oxidative stress.

On the other hand, the study discovered that after receiving an injection of alloxan, the levels of malondialdehyde (MDA), an indication of oxidative stress, were considerably higher in the diabetes group than in the normal group. However, as indicated in Table 7, MDA levels decreased following 8 weeks of administration of *S. oleraceus* extract. These findings corroborate earlier studies showing that phenolic acids can prevent lipid peroxidation and shield tissues from external damage in glycemic rats [47].

## 4. Materials and Methods

The plant material (*Sonchus oleraceus* healthy leaves were in the stage of plant maturity, collected in January 2022) used in our study was grown naturally on a farm at an agricultural research station operated by the Faculty of Agriculture at Moshtohor, Benha University, Egypt. All reagents and standards used in our experiments were purchased from SIGMA-ALDRICH Co. (St. Louis, MO, USA). The experimental animals were healthy one-month-old adult male albino rats (Wister strain) of approximately the same weight from the Energy Authority in INSHAS, Egypt. 

### 4.1. Preparation of Extract for Biological Experiments

Fresh leaves of *S. oleraceus* were cleaned and shade dried. The dried leaves were mechanically ground, immersed in 80% ethanol (1:10) for seven days at room temperature (25–30 °C), and gently mixed each day. The mixture was filtered through filter paper in a Büncher funnel and then concentrated to dryness using a rotary evaporator. Afterward, the extract was dispersed in distilled water with a pH of 6.5 to treat hyperglycemia and given orally to the experimental animal [6].

### 4.2. Preparation of Different Extracts for Determination of Total Phenolic, Total Flavonoid, and Radical–Scavenging Activity by DPPH

The dried leaves were powdered mechanically and extracted by different solvents, including petroleum ether, chloroform, ethyl acetate, 80% ethanol, and distilled water for 15 h for each solvent, using a Soxhlet extractor. The extracts were filtered via a Buchner funnel and evaporated under a vacuum utilizing a rotary vacuum evaporator (N-N series, EYELA, Tokyo, Japan) at 40 °C in the dark [43].

### 4.3. Determination of Total Phenolic Content (TPC)

A UV spectrophotometer (SM1600UV-vis Spectrophotometers, Azzota, Claymont, DE, USA) and a colorimetric oxidation/reduction procedure were used as described by [50], and the total phenol concentration in ethanolic extracts was determined. Folin-Ciocalteu reagent was the oxidizing agent utilized. Briefly, 2.5 mL of Folin-Ciocalteu reagent and 2 mL of Na_2_CO_3_ (75 g L^−1^) were added to 0.5 mL of the diluted extract (10 mg in 10 mL solvent). The samples were incubated for 5 min at 50 °C and then cooled. Distilled water of 0.5 mL was used as the control sample. At 760 nm, the absorptance was measured. Gallic acid equivalent (GAE) measures the TPC.

### 4.4. Determination of Total Flavonoids Content (TFC)

The total flavonoid content was determined using Ordonez et al.’s method [51]. Briefly, 1.5 mL of the 20 g L^−1^ AlCl_3_ ethanolic solutions were added to 0.5 mL of the extract solution (10 mg in 10 mL solvent). One hour after addition, the absorbance at 420 nm was assessed at room temperature, and the yellow color indicates flavonoids. The TFC was expressed as a quercetin equivalent (QE).

### 4.5. DPPH (2,2-Diphenylpicryhydrazyl) Radical–Scavenging Activity 

According to Hanato et al. approach, the obtained extracts’ capacity for electron donation was evaluated by bleaching the DPPH purple solution. To 3 mL of 0.1 mM DPPH dissolved in ethanol, 100 µL of each extract (0.1 mg extract/10 mL solvent) was added [47]. After 30 min at room incubation temperature, the absorbance was compared to a standard at 517 nm [52]. The following formula was used to determine the free radical DPPH’s antioxidant activity percentage:

Antioxidant activity (inhibition)% = [((A(control) − A(sample))/(A(control))] × 100(1)

where A control is the absorbance of the control reaction (100 µL methanol with 3 mL DPPH), and A sample is an absorbance in the plant extract.

### 4.6. HPLC Conditions

An Agilent 1260 series was used for HPLC analysis. Eclipse C18 column (4.6 mm × 250 mm i.d., 5 m) was applied for separation. At a 1 mL/min flow rate, the mobile phase consisted of water (A) and 0.05% trifluoroacetic acid in acetonitrile (B). The mobile phase was programmed in the following order: 0 min (82% A); 0–5 min (80% A); 5–8 min (60% A); 8–12 min (60% A); 12–15 min (82% A); 15–16 min (82% A); and 16–20 min (82% A). At 280 nm, the multi-wavelength detector was monitored. For each of the sample solutions, the injection volume was 5 L. The column temperature was kept constant at 40 °C [53,54].

### 4.7. Experimental Design

A total of 42 one-month-old adult male albino rats (Wister Strain) were housed in a stainless-steel bottom cage with the temperature regulated at 25 ± 2 °C. Rats were housed in a healthy environment for 14 days and provided a basal diet, 50% relative humidity, 12 h light-dark cycle, and free access to water. Starch comprised 65% of the diet, followed by casein, 15%, maize oil, 10%, cellulose, 4% minerals, and 1% vitamins [55]. The Research Ethics Committee approved the experimental animal design numbered (ZU-IACUC/3/F/456/2022).

### 4.8. Administration and Dosage of Sonchus oleraceus Extract (SOE)

The groups were administered 10 mg kg^−1^ glibenclamide, 100 mg kg^−1^, 200 mg kg^−1^, and 300 mg kg^−1^ body weight *Sonchus oleraceus* extract (SOE) [5,6] using a Sondhi needle by gastric gavage. After that, animals were divided into two main groups.

First main group (7 rats): negative control (healthy control) without treatment. Second main group (35 rats): was the diabetic group. Rats were injected with a single dose of alloxan solution of 100 mg kg^−1^ body weight [56,57,58]. After 24 h of alloxan injection, diabetes was confirmed (glucose blood was higher than 200 mg dL^−1^). Rats were left for one week to stabilize diabetes, and then rats were divided into five subgroups (7 rats per each).

The first subgroup (7 rats) was kept as diabetic (positive control) and fed on a basal diet for eight weeks. The second subgroup (7 rats) was fed a basal diet and received orally glibenclamide 10 mg kg^−1^ body weight per day for eight weeks. The third subgroup (7 rats) was fed a basal diet and received orally *S. oleraceus* extract 100 mg kg^−1^ body weight per day for eight weeks. The fourth subgroup (7 rats) was fed a basal diet and received orally *S. oleraceus* extract 200 mg kg^−1^ body weight. The Fifth subgroup (7 rats) was fed a basal diet and orally obtained *S. oleraceus* extract 300 mg kg^−1^ body weight per day for 56 days.

### 4.9. Blood Sampling, Biochemical, and Histological Evaluation 

At the end of the experiment, blood samples were collected and obtained from the retro-orbital plexus veins of the individual rat using fine capillary heparinized tubes. Serum was used to investigate the biochemical parameters, including liver and kidney function tests and serum lipid profile. Activities of liver enzymes, including (ALT) and (AST), as well as the serum total bilirubin (TB), total protein, and serum albumin, were determined corresponding to the methods of [25,59,60,61,62], respectively. The globulin was calculated by subtracting the albumin from the serum total protein. Kidney function parameters, including urea, uric acid, and creatinine, were measured using the [63] method. Lipid profile, including triglycerides (TAG), total cholesterol (TC), high-density lipoprotein cholesterol (HDL), and low-density lipoprotein cholesterol (LDL), was determined according to the methods of [64]. Glucose concentration was determined according to Trinder et al. method [65]. In the present investigation, rats were classified as having severe type-2 diabetes if their blood glucose levels were over 250 mg dL^−1^.

Furthermore, a histological assay was carried out to analyze the pancreatic tissue samples. These tissue samples were obtained from rats in each group and swiftly fixed in 10% neutral buffered formalin to ensure proper preservation. Subsequently, thin paraffin sections were prepared using standard procedures and subjected to microscopic examination. The sections were stained using hematoxylin and eosin stain (H&E) stain, following the guidelines provided by Suvarna et al. in 2013. This histological analysis allowed for a detailed assessment of tissue structures and cellular morphology in the pancreas for each experimental group [66].

## 5. Conclusions

Egyptian *Sonchus oleraceus* ethanolic extracts (SOE) have been studied as potential anti-diabetic treatments with no adverse effects on the liver, kidney functioning, or lipid profile. The negative control group demonstrated a normal histological structure of the pancreas, while the alloxan-induced diabetic rats exhibited degenerative changes. However, the treatment with Glibenclamide and SOE at a dosage of 300 mg kg^−1^ significantly improved the histological features of the pancreas, indicating their potential therapeutic effect in the context of pancreatic health. Moreover, the probable defensive consequence of the SOE against hyperglycemia in albino rats may be attributed to the bio-oxidant status, antioxidant activity, and antihyperglycemic properties of both extracts’ bio-constituents (glycosides, phenolics, and flavonoids), implying the potential utilization of natural antioxidants from herbal sources for diabetes mellitus in vivo, aiming to explore their therapeutic applications while evaluating their long-term effects and safety. 

## Figures and Tables

**Table 1:** The extraction yield and antioxidant potential of *Sonchus oleraceus* extracts.

Sample	Total Extract %	TPC mg GAE/gm	TFC mg QE/gm	DPPH %
E.E.	33.5 ± 0.56	127.66 ± 0.56	74.80 ± 0.55	43.46 ± 0.42
C.F.	4.77 ± 0.65	75.68 ± 0.3	3.52 ± 0.09	16.50 ± 0.47
E.A.	5.20 ± 0.6	100.22 ± 0.46	3.73 ± 0.39	5.04 ± 0.13
P.T.	18.77 ± 0.42	61.25 ± 0.76	5.40 ± 0.52	23 ± 0.91
A.E.	20 ± 0.26	105.5 ± 0.24	37.13 ± 0.75	37.82 ± 0.21

SD: Standard deviation, *n* = 3; E.E. ethanolic extract; C.F. chloroformic; E.A. ethyl acetate; P.T. Petroleum ether; A.E. aqueous extract; DPPH 2,2-diphenyl-1-picrylhydrazyl.

**Table 2:** Identification of some antioxidant components of leaves ethanolic extract by HPLC.

Leaves Components	RT (Min)	Concentration (%)
Phenolic compounds		
Chlorogenic acid	3.914	28.02
Catechin	4.12	37.39
Methyl gallate	5.099	0.31
Coffeic acid	5.460	0.81
Syringic acid	5.822	0.47
Flavonoid compounds		
Vanillin	8.845	16.58
Ferulic acid	9.421	10.02
Naringenin	10.092	11.30
Quercetin	12.840	4.29
Cinnamic acid	14.037	0.22
Hesperetin	15.729	24.79

**Table 3:** Comparison between the different studied groups according to effect on body weights gain.

Groups		Initial Body Weight (g)	Final Body Weight (g)	Change Body Weight Gain	
				(g)	(%)
1	Control Negative	104.7 ^b^ ± 8.1	240.7 ^a^ ± 23.5	136 ^a^ ± 29.6	131.6 ^a^ ± 36.2
2	Control Positive Diabetic (100 mg kg^−1^ Alloxan)	174.3 ^a^ ± 15	196.3 ^a^ ± 2.3	22 ^b^ ± 17	13.4 ^b^ ± 11.1
3	Diabetic + 10 mg kg^−1^ GLI	153 ^a^ ± 15.4	237.7 ^a^ ± 22.5	84.7 ^a^ ± 34.2	57 ^b^ ± 27.3
4	Diabetic + 100 mg kg^−1^ SOE	149.7 ^a^ ± 9.7	268.3 ^a^ ± 27.1	118.7 ^a^ ± 36.8	80.6 ^ab^ ± 30.5
5	Diabetic + 200 mg kg^−1^ SOE	154 ^a^ ± 14.4	256.3 ^a^ ± 48.7	102.3 ^a^ ± 35.5	65.6 ^ab^ ± 18.4
6	Diabetic + 300 mg kg^−1^ SOE	155.7 ^a^ ± 12.1	265.7 ^a^ ± 28.2	110 ^a^ ± 31.6	71.5 ^ab^ ± 23.9

SD: Standard deviation; *n* = 7 for each group SOE: *Sonchus oleraceus* Extract; GLI: Glibenclamide. Note: Different letters (a and b) refer to significant differences (*p* < 0.05) within the values of each column.

**Table 4:** Effect of SOE extract and GLI treatment on blood glucose in negative control and positive control (diabetic) groups.

G.	Treatments	Glucose (mg dL^−1^) Ratio		
		Zero Point	2 Days	56 Day
1	Control Negative	128 ^a^ ± 9.8	120.3 ^b^ ± 1.5	104.3 ^b^ ± 14.4
2	Control Positive Diabetic (100 mg kg^−1^ Alloxan)	96.3 ^a^ ± 20.1	370 ^a^ ± 115.3	405 ^a^ ± 158.3
3	Diabetic + 10 mg kg^−1^ GLI	118 ^a^ ± 6.1	314.7 ^ab^ ± 115.5	184.3 ^b^ ± 93.4
4	Diabetic + 100 mg kg^−1^ SOE	120 ^a^ ± 5	349.7 ^ab^ ± 64.5	151 ^b^ ± 16.4
5	Diabetic + 200 mg kg^−1^ SOE	105.7 ^a^ ± 12.5	276.7 ^ab^ ± 64	138.7 ^b^ ± 27.2
6	Diabetic + 300 mg kg^−1^ SOE	101.7 ^a^ ± 13.3	318.3 ^ab^ ± 28.4	103.7 ^b^ ± 9.3

SD: Standard deviation *n* = 7 for each group. SOE: Sonchus oleraceus Extract; GLI: Glibenclamide. Note: Different letters (a and b) refer to significant differences (*p* < 0.05) within the values of each column.

**Table 5:** Effect of treatment with SOE extract and GLI on lipid profile.

G.	Treatments	Total Cholesterol (mg dL^−1^)	Triglyceride (mg dL^−1^)	HDLC (mg dL^−1^)	LDLC (mg dL^−1^)
1	Control Negative	123.7 ^bc^ ± 3.2	124.3 ^c^ ± 5	54 ^a^ ± 3.6	57 ^b^ ± 2
2	Control Positive Diabetic (100 mg kg^−1^ alloxan)	133 ^a^ ± 3.6	195.7 ^a^ ± 3.1	46.7 ^ab^ ± 2.1	66.3 ^a^ ± 4.7
3	Diabetic + 10 mg kg^−1^ GLI	122.3 ^bc^ ± 2.5	143.3 ^b^ ± 4.2	45 ^ab^ ± 3	53 ^b^ ± 4.4
4	Diabetic + 100 mg kg^−1^ SOE	121 ^c^ ± 2.6	203 ^a^ ± 6.1	41 ^b^ ± 3.6	40.7 ^c^ ± 2.1
5	Diabetic + 200 mg kg^−1^ SOE	129 ^ab^ ± 1	117.7 ^c^ ± 2.5	53.3 ^a^ ± 4.9	54 ^b^ ± 2.6
6	Diabetic + 300 mg kg^−1^ SOE	124.7 ^bc^ ± 1.5	98.7 ^d^ ± 1.5	44.7 ^ab^ ± 3.1	66 ^a^ ± 1

SD: Standard deviation *n* = 7 for each group. SOE: *Sonchus oleraceus* Extract; GLI: Glibenclamide. Note: Different letters (a, b, c and d) refer to significant differences (*p* < 0.05) within the values of each column.

**Table 6:** SOE extract and glibenclamide therapy on hepatic and renal functions in diabetic rats compared to GLI, negative and positive control (diabetic) groups.

		Liver Function						Kidney Function		
G.	Treatment	AST (U L^−1^)	ALT (U L^−1^)	T. Protein (mg dL^−1^)	Albumin (mg dL^−1^)	Globulin (mg dL^−1^)	Bilirubin (mg dL^−1^)	Urea (mg dL^−1^)	Uric Acid (mg dL^−1^)	Creatinine (mg dL^−1^)
1	Control Negative	61.2 ^b^ ± 1.6	68.3 ^b^ ± 1.5	6.4 ^a^ ± 0.4	4.5 ^a^ ± 0.5	2 ^ab^ ± 0.2	0.13 ^ab^ ± 0.01	36 ^b^ ± 4.36	2.9 ^ab^ ± 0.2	1.37 ^bc^ ± 0.38
2	Control Positive Diabetic (100 g kg^−1^ alloxan)	82.0 ^b^ ± 3.0	73.3 b ± 2.9	6.4 ^a^ ± 0.2	3.9 ^a^ ± 0.1	2.3 ^ab^ ± 0.5	0.05 ^b^ ± 0.04	52 ^a^ ± 2	3.3 ^a^ ± 0.6	1.87 ^ab^ ± 0.80
3	Diabetic + 10 mg kg^−1^ GLI	51.3 ^c^ ± 1.5	65.1 ^b^ ± 1	6.3 ^a^ ± 0.6	4.2 ^a^ ± 0.6	2.6 ^a^ ± 0.5	0.04 ^b^ ± 0.04	35 ^b^ ± 2	2.5 ^b^ ± 0.4	1.23 ^bc^ ± 0.16
4	Diabetic + 100 mg kg^−1^ SOE	118 ^a^ ± 2	148 ^a^ ± 2	6.4 ^a^ ± 0.4	3.8 ^a^ ± 0.3	2.2 ^ab^ ± 0.3	0.17 ^a^ ± 0.05	43 ^b^ ± 3	3.6 ^a^ ± 0.3	2.20 ^a^ ± 0.44
5	Diabetic + 200 mg kg^−1^ SOE	42 ^d^ ± 2.6	54.3 ^c^ ± 2.1	6 ^a^ ± 0.5	4.5 ^a^ ± 0.5	1.5 ^b^ ± 0.4	0.14 ^ab^ ± 0.05	43.7 ^ab^ ± 3.1	2.8 ^ab^ ± 0.4	1.90 ^ab^ ± 0.40
6	Diabetic + 300 mg kg^−1^ SOE	55 ^c^ ± 2	64.7 ^b^ ± 3.2	6.1 ^a^ ± 0.2	4.7 ^a^ ± 0.4	1.9 ^ab^ ± 0.2	0.09 ^ab^ ± 0.02	25 ^c^ ± 4	2.6 ^ab^ ± 0.5	0.96 ^c^ ± 0.11

SD: Standard deviation, *n* = 7 for each group. SOE: *Sonchus oleraceus* Extract; GLI: Glibenclamide. Note: Different letters (a, b, c, and d) refer to significant differences (*p* < 0.05) within the values of each column.

**Table 7:** Effect of treatment with SOE and glibenclamide on the antioxidant parameter in diabetic rats compared to glibenclamide (GLI) and negative control and positive control (diabetic) groups.

G.	Treatments	MDA (mM L^−1^)	SOD (U L^−1^)	GSH (U L^−1^)	CAT (U L^−1^)	(GSTs) (U L^−1^)
1	Control Negative	10 ^b^ ± 1	43 ^ab^ ± 2	66 ^a^ ± 1	77 ^a^ ± 2.6	99 ^a^ ± 1
2	Control Positive Diabetic (100 mg kg^−1^ alloxan)	18.7 ^a^ ± 1.5	34.7 ^bc^ ± 2.1	42.3 ^d^ ± 2.5	62.3 ^b^ ± 2.5	76 ^c^ ± 2
3	Diabetic + 10 mg kg^−1^ GLI	12.7 ^b^ ± 2.5	31.3 ^c^ ± 3.2	52.7 ^c^ ± 2.5	65 ^b^ ± 4.4	86 ^b^ ± 4.4
4	Diabetic + 100 mg kg^−1^ SOE	13 ^b^ ± 2.6	42 ^abc^ ± 2.6	56.3 ^bc^ ± 1.5	67 ^b^ ± 2	93 ^ab^ ± 4.4
5	Diabetic + 200 mg kg^−1^ SOE	13 ^b^ ± 2	44.3 ^ab^ ± 4	55 ^c^ ± 3	69.7 ^ab^ ± 2.5	92 ^ab^ ± 2
6	Diabetic + 300 mg kg^−1^ SOE	10.7 ^b^ ± 2.1	45.7 ^a^ ± 7.4	61 ^ab^ ± 1	69 ^b^ ± 2	93 ^ab^ ± 2

SD: Standard deviation *n* = 7 for each group. Note: Different letters (a, b, c, and d) refer to significant differences (*p* < 0.05) within the values of each column.

## Data Availability

The data presented in this study are available in Supplementary Materials.

## Supplementary Materials

The following supporting information can be downloaded at: https://www.mdpi.com/article/10.3390/molecules28176389/s1. Figure S1: HPLC identification of several antioxidant components in the ethanolic leaf extract.

## Abbreviations

SOE: *Sonchus oleraceus* extract; DPPH: 1,1-diphenyl−2-picrylhydrazyl; GAE: gallic acid equivalent; QE: quercetin equivalent; GLI: glibenclamide; LDLC: low-density lipoprotein; HDLC: high-density lipoprotein; HPLC: high performance chromatography; ALT: alanine transaminase; AST: aspartate amino ferase; SOD: Superoxide dismutase; GSH: glutathione reductase; GSTs: glutathione-S-transferases; CAT: Catalase; MDA: Malondialdehyde; TPC: Total Phenolic Content; TFC: Total Flavonoid Content.
