# Supplementary material for: Phenolic Profiles, Antihyperglycemic, Anti-Diabetic, and Antioxidant Properties of Egyptian Sonchus oleraceus Leaves Extract: An In Vivo Study
Source: Molecules. 2023 Sep 1;28(17):6389. doi: 10.3390/molecules28176389 (PMC10489745; doi:10.3390/molecules28176389)
Supplement: Supplementary file 1 [file molecules-28-06389-s001.zip › molecules-2561956-supplementary.pdf]

molecules

MDPI

Article

Phenolic Profiles, Antihyperglycemic, Anti-diabetic, and Antioxidant Properties of Egyptian *Sonchus oleraceus* Leaves Extract: An In Vivo Study

Nesrein S. Salim [1], Mohamed Abdel-Alim [1], Huda E. M. Said [2] and Mohamed F. Foda [1,3,*]

[1] Department of Biochemistry, Faculty of Agriculture, Benha University, Moshtohor 13736, Egypt; nesreen.salem@fagr.bu.edu.eg (N.S.S.); mohamed.abdelalem@fagr.bu.edu.eg (M.A.-A.)
[2] Clinical Pathology Department, Faculty of Medicine, Zagazig University, Zagazig 44519, Egypt; hesaid@medicine.zu.edu.eg
[3] College of Life Science and Technology, Huazhong Agricultural University, Wuhan 430070, China
* Correspondence: m.frahat@fagr.bu.edu.eg

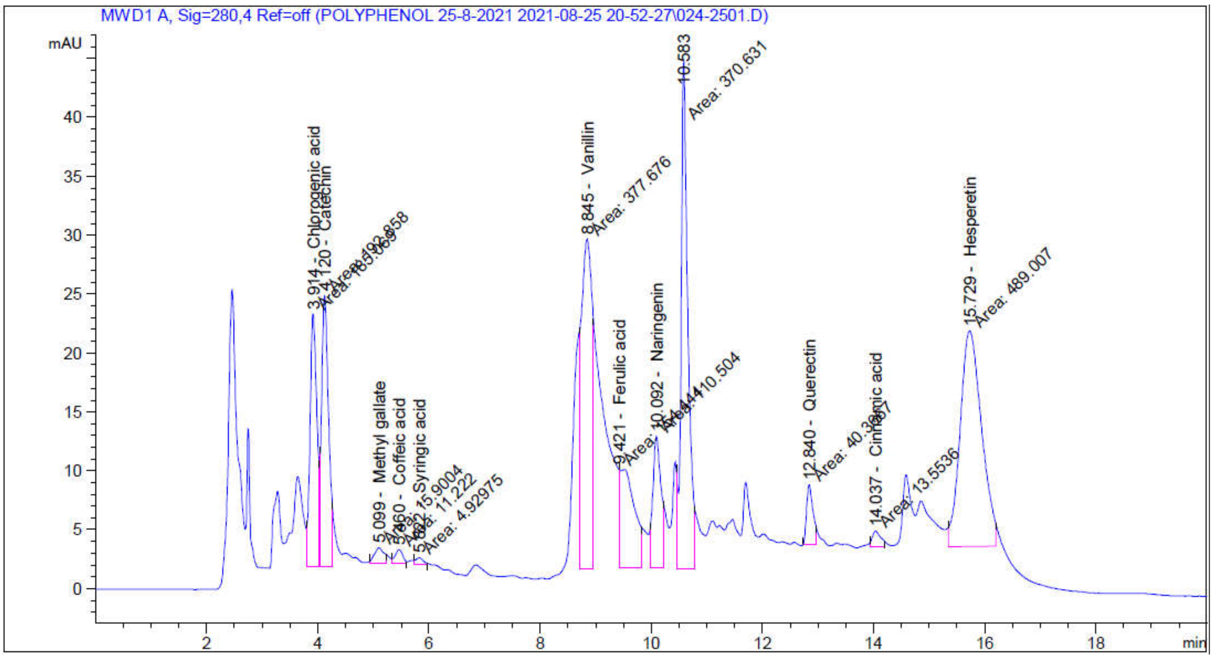

Figure S1: HPLC identification of several antioxidant components in the ethanolic leaf extract.
